# Pain Intensity and Health Service Utilization in United States Adults with Pain: A Cross-Sectional Database Analysis

**DOI:** 10.3390/healthcare13141678

**Published:** 2025-07-11

**Authors:** David R. Axon, Blair Jensen, Jordanne Koulong Kuemene, Mason Leech, Estabraq Mahmood

**Affiliations:** Department of Pharmacy Practice & Science, R. Ken Coit College of Pharmacy, The University of Arizona, 1295 N. Martin Ave., Tucson, AZ 85721, USA; blairjensen@arizona.edu (B.J.); jkoulongkuemene@arizona.edu (J.K.K.); masonsleech@arizona.edu (M.L.); estabraqmahmood@arizona.edu (E.M.)

**Keywords:** pain, health service utilization, emergency room visits, inpatient visits, office-based visits, outpatient visits, United States adults

## Abstract

Background: Pain is a common, often debilitating ailment that may necessitate considerable health service utilization. However, there is a need to assess the associations of pain intensity and other variables with health service utilization among United States adults who have pain. Methods: This cross-sectional database analysis made use of the Medical Expenditure Panel Survey full-year consolidated data file and included United States adults (≥18 years) who have pain. The dependent variables consisted of four health service utilization variables, which included the number of emergency room visits, inpatient discharges, office visits, and outpatient visits in 2021. The number of visits or discharges were categorized as either ≥1 or 0. The independent variable was pain intensity (extreme, quite a bit, moderate, or little pain). Other variables analyzed included age, race, ethnicity, sex, marriage, education, employment, income, insurance, chronic conditions, limitations, exercise, smoking, physical health, and mental health. Chi-squared tests compared differences between pain intensity groups, and multivariable logistic regression models assessed the associations of pain intensity and other variables with each of the four health service utilization variables. The analysis was weighted for national estimates. The significance (alpha) level was 0.05. Results: This analysis included 6280 adults, representing 89,314,769 United States adults with pain. In the multivariable analyses, there were statistically significant associations for extreme pain (odds ratio = 1.72, 95% confidence interval = 1.27–2.33), quite a bit of pain (odds ratio = 1.75, 95% confidence interval=1.37–2.24), and moderate pain (odds ratio = 1.28, 95% confidence interval = 1.02–1.60) versus little pain with emergency room visits, extreme pain (odds ratio = 2.10, 95% confidence interval = 1.44–3.08) and quite a bit of pain (odds ratio = 1.66, 95% confidence interval = 1.21–2.28) versus little pain with inpatient discharges, and quite a bit of pain (odds ratio = 1.47, 95% confidence interval = 1.03–2.11) versus little pain with office visits. There was no correlation between pain intensity levels and outpatient visits. In addition, several other variables were associated with various health service utilization variables. Conclusions: This database analysis discovered greater pain intensity levels were often correlated with increased health service utilization, including more emergency room, inpatient, and office visits. These findings may inform the development of targeted interventions for people with specific characteristics. Further work is needed to implement initiatives that optimize health service utilization and ultimately improve health outcomes for United States adults who have pain.

## 1. Introduction

Pain is depicted as an unpleasant and often unwanted physical and emotional encounter that is related to actual or potential damage to bodily tissues [1]. Pain perception involves the central and peripheral nervous systems and can be subjective. Thus, appropriate pain assessment remains a challenging barrier to treatment [2]. There are several classifications and types of pain. These include nociceptive, inflammatory, and pathological pain [3], as well as acute pain (lasting less than three months) or chronic pain (persisting for more than three months without relief) [4]. Chronic pain may develop from a prolonged exposure to inflammatory and neuropathic responses to painful stimuli, which leads to alterations in pain pathways and ultimately peripheral and central nervous system sensitization. Chronic pain can also occur from poorly managed acute pain [5]. 

Pain is a worldwide problem affecting an estimated one-in-five adults [6] and is often insufficiently treated in healthcare settings [7]. Pain poses substantial challenges to individuals, health systems, and society [1]. The effects of pain on society consist of numerous consequences such as depression, inability to work, and disrupted social relationships [6]. If neglected, pain can affect one’s behavior, ability to perform physical activity, personal interactions, and overall quality of life [8]. Pain continues to pose a national health problem within the United States due to the extent to which it affects the population. Affecting over 50 million United States adults in 2022 [9], pain impacts disability, morbidity, and mortality and places a considerable strain on the health system [1]. Alongside the economic impact of lost wages, managing pain has surpassed spending on cancer, diabetes, and heart disease [1,9]. 

People who have pain utilize several approaches to manage their pain, including medications and health services, which can be burdensome [10]. Careful consideration must mitigate the optimum agent, regimen, duration, and administration to control each person’s unique pain experience while also avoiding unnecessary titrations, evaluating drug interactions, and avoiding overdose [11]. Tailoring regimens is important for warranting adequate quality-of-life and avoiding financial burden for both patients and institutions [12]. 

Individuals with pain may engage with various health services to help manage their pain. For instance, they may visit their primary care provider for regular consultations or attend outpatient clinics for assessments. In cases where the person has extreme pain, they may visit the emergency room or even be admitted to hospital. Health services should be pillars in effectively managing patient pain regimens [13]. Yet some health services fail to optimize and may even exacerbate pain [13]. More sophisticated facilities such as skilled nursing facilities also struggle with pain management [14]. For instance, one report states there were over 20% composite readmissions and death rates within 30 days of discharge in patients reported to have moderate to severe pain during their admission [14]. Furthermore, data demonstrates patients are more likely to use health services in greater volume when experiencing daily pain, especially when it is linked to other comorbid chronic conditions. Multimodal pain entities, including pain specialists, psychologists, and internists, show an almost doubled average number of visits per month for people categorized with severe daily pain. Hospital admission and emergency room visits were associated with >300% and >350% increases for patients indicated to have severe pain, respectively [15]. Research has also shown that demographic variables such as age and gender can impact healthcare service utilization among individuals with pain. For instance, older adults may require a greater frequency of healthcare visits because of ongoing medical care for age-related health conditions [16]. 

Given the established impact of pain on individuals and society and the complexity of pain management approaches in the health system, there is interest in better understanding the association between pain intensity and health service utilization. There is also interest in better understanding other personal characteristics that may be associated with health service utilization among people with pain. For instance, it would be useful to know whether greater levels of pain intensity are associated with health service utilization, or whether certain personal characteristics of people with pain are associated with health service utilization. This information may help identify disparities between pain intensity or personal characteristics and health service utilization. In turn, this may help inform the development of more targeted policies or services to help these individuals better manage their health. The objective of this paper was to investigate the correlation between pain intensity and other variables with health service utilization, including emergency room visits, inpatient discharges, office visits, and outpatient visits, among United States adults who have pain. This article is a revised and expanded version of a paper entitled “Association between pain intensity and health service utilization (emergency room visits, inpatient discharges, office visits, outpatient visits) among adults with pain in the United States”, which was presented at the American Society for Health-System Pharmacists Midyear Clinical Meeting in New Orleans, Louisiana, United States on 8 December 2024 [17].

## 2. Materials and Methods

This was a cross-sectional database analysis that employed the Medical Expenditure Panel Survey data. The Medical Expenditure Panel Survey is a nationwide United States survey of households that seeks to capture a multitude of data on health or medical characteristics, including expenditures and utilization of services. The Medical Expenditure Panel Survey also collects many complementary variables that can be used to describe the sample demographics. The Medical Expenditure Panel Survey uses the sampling frame of the National Health Interview Survey and includes weighting variables that can be employed when analyzing the study sample to report estimates that are nationally representative of the United States civilian, non-institutionalized population. Participants are surveyed several times throughout the year to collect self-reported data in the Medical Expenditure Panel Survey household component, which are then enhanced with data from the Medical Expenditure Panel Survey medical provider component (data from medical providers) and the Medical Expenditure Panel Survey insurance component (data from health insurers). The Medical Expenditure Panel Survey staff quality check and collate the data into files that are available for public use, one of which is the 2021 full-year consolidated data file that was utilized in this analysis. This person-level dataset contained data for 28,336 United States individuals [18,19].

Eligibility criteria for this study included United States adults who were alive throughout 2021 and who had pain in the previous four weeks. This study had four outcome variables that represented health service utilization: (1) number of emergency room visits, (2) inpatient discharges, (3) office visits, and (4) outpatient visits in the calendar year (2021). These were categorized for analysis as ≥1 visit/discharge or 0 visits/discharges to determine who had used the health service at least once versus those who had not used the health service at all during 2021. The key independent variable was pain intensity in the past four weeks, which was derived based on responses to the following self-reported survey item: “During the past four weeks, how much did pain interfere with your normal work (including both work outside the home and housework)?” Survey response options included extremely, quite a bit, moderately, a little bit, and not at all. These were categorized for analysis as extreme, quite a bit, moderate, or little. Those who did not report that they had pain in the past four weeks, or did not respond to the pain item, were not included in this study. Demographic variables, which later served as covariates in the multivariable analysis, included age (≥70, 60–69, 50–59, 40–49, 30–39,18–29 years), race (white, others), being Hispanic (yes, no), sex (male, female), marriage (married, not married), education (≤high school, >high school), employment (employed, unemployed), income (low, middle, high), insurance (private, public, none), the number of chronic conditions, including hypertension, coronary heart disease, angina, heart attack, other heart disease, stroke, emphysema, chronic bronchitis in the last 12 months, high cholesterol, cancer, diabetes, joint pain in the last 12 months, arthritis, and asthma (0–1, 2–3, 4–5, 6+ conditions), any limitations (which includes any activity of daily living limitations, instrumental activity of daily living limitations, functional limitations, work limitations, sight limitations, or hearing limitations; yes, no), exercise (defined as participating in at least 30 min of moderate to vigorous intensity exercise at least five times per week; yes, no), being a current smoker (yes, no), health (excellent/very good, good, fair/poor), and mental health (excellent/very good, good, fair/poor). Further details of the variables used in the analysis are provided in Appendix A.

Data analysis was performed using SAS software (version 9.4, SAS Institute Inc., Cary, NC, USA) and the SAS survey procedures. The eligibility criteria were applied and any individuals with missing data were excluded from the analysis. The appropriate Medical Expenditure Panel Survey sampling weight variable was used to calculate estimates that were nationally representative of the eligible sample. The Medical Expenditure Panel Survey cluster and strata variables were employed to manage the structure of complex survey datasets. Chi-squared tests were used to test for differences between the pain intensity groups. A univariate and multivariable (adjusting for all previously described demographic variables) binary logistic regression model was used for each of the four health service utilization variables (emergency room visits, inpatient discharges, office visits, outpatient visits). The assumptions of logistic regression (namely independent observations and no multicollinearity) were assessed and met satisfactorily. An alpha value of 0.05 was selected a priori as the statistical significance threshold. All individuals who participate in the Medical Expenditure Panel Survey provide informed consent to participate. The University of Arizona Institutional Review Board approved this study (approved 22 February 2023, #00002551). This study adhered to the principles outlined in the Declaration of Helsinki. 

## 3. Results

**Figure 1:**
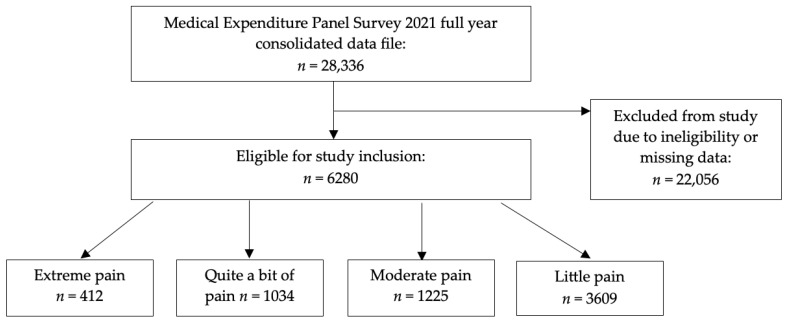
Participant inclusion flowchart.

Figure 1 presents the participant inclusion flowchart. Overall, 6280 adults who had pain were included in this analysis. This represented a weighted population of 89,314,769 adults with pain across the US. The majority of these had little pain (*n* = 3609; weighted *n* = 55,805,431). The remainder had moderate pain (*n* = 1225; weighted *n* = 16,203,128), quite a bit of pain (*n* = 1034; weighted *n* = 12,473,024), and extreme pain (*n* = 412; weighted *n* = 4,833,186).

Table 1 presents the characteristics of United States adults stratified by pain intensity in the weighted study population. The majority of the population were white (79.4%), non-Hispanic (88.2%), female (55.1%), married (53.2%), had more than high school education (57.1%), employed (53.2%), had private insurance (60.1%), no limitations (58.7%), did not frequently exercise (54.4%), and were non-smokers (85.6%). There was no overall majority for the following variables, although the most common groups were age ≥ 70 years (24.2%), high income (41.0%), 2–3 chronic conditions (35.9%), very good/excellent physical health (38.6%), and very good/excellent mental health (47.1%). There were statistically significant differences between the four pain intensity levels for most demographic variables with the exception of race and Hispanic status.

Table 2 presents the unadjusted correlations of pain intensity with emergency room visits, inpatient discharges, office visits, and outpatient visits among United States adults who had pain. Compared to those with little pain, United States adults who had extreme pain or quite a bit of pain were associated with higher odds of health service utilization for all four categories (emergency room visits, inpatient discharges, office visits, and outpatient visits). Those with moderate pain were associated with higher odds of health service utilization for emergency room visits and outpatient visits only. 

Table 3 presents the adjusted correlations of pain intensity and other demographic variables with emergency room visits, inpatient discharges, office visits, and outpatient visits among United States adults who had pain. United States adults who had extreme pain, quite a bit of pain, or moderate pain were associated with higher odds of emergency room visits compared to those with little pain. United States adults who had extreme pain or quite a bit of pain were associated with higher odds of inpatient discharges compared to those with little pain. Only United States adults who had quite a bit of pain were associated with higher odds of office visits compared to those with little pain, while there was no association for any level of pain intensity and outpatient visits. 

In addition, age ≥ 70 years, 60–69 years, and 50–59 (compared to 18–29) years were each associated with lower odds of emergency room visits, while age ≥ 70 (compared to 18–29) years was associated with higher odds of inpatient discharges, office visits, and outpatient visits. White (compared to other) race was associated with higher odds of office visits. Being Hispanic (compared to not Hispanic), being male (compared to female), and having high school education or less (compared to more than high school education) were each associated with lower odds of office visits and outpatient visits. Being employed (compared to unemployed) was associated with higher odds of office visits. Having a low (compared to high) income was associated with lower odds of office visits, while low and middle (compared to high) income was associated with lower odds of outpatient visits. Having private or public (compared to no) insurance and having a limitation (compared to no limitation) were each associated with higher odds of using all four health services. Having fewer chronic conditions was associated with progressively lower odds of emergency room visits, inpatient discharges, and outpatient visits, while having 0–1 (compared to ≥6) chronic conditions was only associated with lower odds of office visits. Being a current smoker (compared to nonsmoker) was associated with higher odds of emergency room visits, yet lower odds of office or outpatient visits. Poor/fair or good (compared to excellent/very good) health was associated with higher odds of emergency room and outpatient visits, while only poor/fair (compared to excellent/very good) health was associated with higher odds of inpatient discharges and only good (compared to excellent/very good) health was associated with higher odds of office visits. Poor/fair (compared to excellent/very good) mental health was associated with lower odds of inpatient discharges.

## 4. Discussion

The findings from the key independent variable (i.e., pain intensity) in this study indicate that United States adults who have extreme pain, quite a bit of pain, or moderate pain were associated with higher odds of emergency room visits compared to those who have little pain, and United States adults who have extreme pain or quite a bit of pain were associated with higher odds of inpatient discharges compared to those who have little pain. Meanwhile, only United States adults who have quite a bit of pain were associated with higher odds of office visits compared to those who have little pain, and there was no association for any level of pain intensity and outpatient visits. 

These findings help us understand how people with pain are currently using health services based on their level of pain. Reasons to directly explain these findings are not forthcoming in the extant literature, though it is possible to offer some potential explanations. For instance, if a person has little or moderate pain, it may be the case that they are able to satisfactorily manage their pain through self-care and/or over-the-counter remedies, negating the need to interact with the healthcare system. It is perhaps logical that a person with a greater level of pain would seek emergency room services, as compared to someone with a lower level of pain. Likewise, it is also more likely that a person who has extreme or quite a bit of pain would have an inpatient hospital stay relative to those with less pain. When considering the findings for office visits, it may be the case that quite a bit of pain represents the optimal level where patients seek support from their healthcare provider’s office. That is, if their pain intensity is greater than quite a bit of pain (e.g., extreme pain), then an office visit will not meet the needs of the patient expeditiously enough and they may instead seek services from the emergency room or hospital. Conversely, if their pain intensity is less than quite a bit of pain (e.g., moderate or little pain), then an office visit may not be necessary if the patient is able to manage their pain themselves. With regard to the findings for outpatient services, it may be the case that patients do not routinely seek outpatient services specifically for pain—rather, they use self-care or visit their provider’s office for lesser levels of pain, or emergency room and hospitalizations for more intense pain. It may also be the case that primary care offices or emergency rooms are more easily accessible to people with pain relative to outpatient facilities. 

This study also identified additional variables (beyond the key independent variable) that showed a statistical association with pain intensity among United States adults who have pain that warrant discussion. For instance, people aged ≥ 50 (age groups ≥ 70, 60–69, and 50–59) were associated with lower odds of emergency room visits relative to those aged 18–29. This is contradictory to some of the recent literature. For instance, among United States adults, data from the 2019 and 2020 National Hospital Ambulatory Medical Care Survey demonstrated that emergency room visit rates were greatest among United States adults ≥ 75 years of age [20]. However, this was among United States adults regardless of pain status and occurred during the COVID-19 pandemic, which may represent atypical healthcare service utilization. In another study using data from the 2014 to 2017 National Hospital Ambulatory Medical Care Survey of United States adults aged > 60 years of age, emergency room visit rates increased with age [21]. The same study showed that the proportion of United States adults aged ≥ 60 years of age who were admitted to hospital following an emergency room visit also increased with age [21]. A literature review suggests that frequent emergency room use is complex and depends on how the data are interpreted—overall, frequent emergency room users were more likely to be <65 years old, yet when the data are disaggregated, frequent emergency room users were more likely to be 25–44 years old and >65 years old [22]. In the current study, there may be other factors, such as mobility issues, caregiver support, or transportation limitations, that inhibit emergency room visits among older adults. Further work to explore emergency room use specifically among United States adults with pain may be necessary to better understand these patterns of healthcare service use. Furthermore, it was unsurprising that the current study found older adults (i.e., those aged ≥ 70 years) were associated with higher odds of inpatient discharges, office visits, and outpatient visits relative to those aged 18–29. Older adults typically have multiple chronic conditions to manage and begin to experience functional decline that necessitates greater interaction with the healthcare service [23,24]. For instance, one study found that approximately 20% of hospitalized adults aged ≥ 65 had severe or moderate pain [25]. The World Health Organization suggests the need to improve healthcare integration for older people [26]. This may benefit older United States adults with pain in the current study who used a variety of healthcare services. 

The current study found white (compared to other) race was associated with higher odds of office visits, while Hispanic (compared to non-Hispanic) ethnicity status was associated with lower odds of office visits and outpatient visits. Unacceptable racial and ethnic disparities still exist in healthcare [27], which appears to be supported by the findings of the current study and other recent publications. A longitudinal study of National Health Interview Survey data from 1999 to 2018 indicated that there has been an increase in barriers to timely medical care for all population groups but noted that the disparity between minority races and ethnicities increased relative to white non-Hispanic people [28]. Another study, using several datasets such as the Medical Expenditure Panel Survey, Medicare Current Beneficiary Survey, National Health Interview Survey, and Disease Expenditure Project, found that the White population received approximately 15% more spending on ambulatory care than the all-population mean. Other races typically had lower spending on overall healthcare costs, for instance; Black non-Hispanic patients received approximately 26% less spending on ambulatory care. Yet, Black non-Hispanic patients received approximately 19% and 12% more spending on inpatient and emergency room care, respectively, compared to the total population [29]. Meanwhile, Hispanic patients received approximately 33% less spending on ambulatory care than the all-population mean [29]. Further research on racial and ethnic disparities in the United States healthcare system is therefore warranted [30]. 

The current study showed that males were associated with lower odds of using office or outpatient services compared to females. Many studies over time have shown that males use the healthcare system less frequently than females [31,32,33,34,35,36]. There are well-documented reasons for this, including masculine gender norms, negative perceptions of the healthcare system or healthcare professionals, and financial constraints [37]. In addition, sex differences in pain are known to exist, with females typically at higher risk for having pain and experiencing more severe pain than males [38,39,40]. The findings of the current study therefore affirm existing knowledge of sex or gender differences in healthcare system use and pain perception. These findings also offer support for the need to better differentiate the design of pain management programs for males and females, given that their experiences with pain and engagement with the health system are different. The findings also reiterate the need for greater effort to encourage males to seek primary care services when needed. 

Having less than high school (compared to more than high school) education was associated with lower odds of office visits and outpatient visits in the current study. This is interesting given that previous work has suggested individuals with lower educational attainment have a greater need, or risk, for healthcare services [41]. Likewise, another Medical Expenditure Panel Survey study of United States adults found that lower educational status was associated with poorer health [42], while a further study specifically among older United States adults who have pain also found lower educational status was associated with poorer health [43]. The relationship between educational status and need for healthcare service use, as well as actual healthcare service use, is complex and may warrant further investigation. 

Employment, income, and insurance status were each associated with healthcare service utilization. It is logical that individuals who are employed have the financial means or health insurance to attend a physician’s office, while having a higher income also provides the financial means to utilize healthcare services. The finding that having private or public (compared to no) insurance was associated with higher odds of using all four health services demonstrates the importance of health insurance in being able to access health services [44], though some recent research suggests public health insurance may be a more cost-effective option than private insurance [45,46]. 

In the current study, poor/fair or good (compared to excellent/very good) health was associated with higher odds of emergency room and outpatient visits, while only poor/fair (compared to excellent/very good) health was associated with higher odds of inpatient discharges. These are perhaps logical findings; one might expect that people who have used the emergency room, outpatient clinic, or had a hospital stay would consider their health to be poorer than those who have not had to use these services. The finding that only those with good (compared to excellent/very good) health was associated with higher odds of office visits may have a more complex interpretation; it is possible that these people are not in perfect health, but any conditions they do have are adequately managed through preventive healthcare services obtained through regular physician’s office visits. Meanwhile, those with poorer health do not visit their healthcare provider as frequently, perhaps due to the lack of financial or other resources or a lack of willingness. Instead, it appears people with poorer health seek emergency room visits when necessary. Of note, there was no association between mental health status and healthcare service utilization in the current study, except that individuals with poor/fair (versus excellent/very good) mental health were associated with lower odds of inpatient discharges. Reasons to explain this are not clear in the literature. As society increases its focus on mental health, further research may be warranted into the healthcare utilization of United States adults with pain and poorer mental health specifically. Related to health status, it was unsurprising that having fewer chronic conditions was associated with lower odds of healthcare service utilization, while having some type of limitation was associated with higher odds of healthcare service utilization in the current study. The finding that current smokers (compared to nonsmokers) were associated with higher odds of emergency room visits, yet lower odds of office or outpatient visits, may be because smokers have less concern for preventive health (that is, fewer office visits) and only seek healthcare services in an emergency (that is, more emergency room visits). 

The Centers for Disease Control and Prevention recommend people with pain work with their healthcare provider to better understand and manage their pain [47]. For instance, the provider can explain the different management strategies available, including opioid medications, non-opioid medications, behavioral treatments, and various other non-pharmacological therapies [11,47]. The provider can also help the patient understand the risks of any therapies and when to engage with the health system. The findings of the current study show that while some people with pain are using the healthcare system, others are not. Furthermore, some individuals may be using the healthcare system when their pain has progressed, e.g., individuals who used the emergency room. Greater clinical care and improved health policy may help individuals manage their pain more effectively. These findings suggest that more effort could be made to encourage preventative or early intervention encounters with primary care providers through office visits, in an attempt to reduce the amount of emergency room or inpatient discharges. 

When reviewing the findings of this study, readers may find it useful to consider the findings of this study from a clinical perspective, not just a statistical perspective. For instance, there are cases where the odds ratio was statistically significant but the confidence interval for the odds ratio was close to 1. One example of this is the association between moderate pain and emergency room visits, where the odds ratio was 1.28 with a confidence interval of 1.02–1.60. In such cases, the actual clinical effect may be relatively minor despite the statistical result. The statistical effect may be further compounded by the large sample size included in this analysis. Therefore, the findings of this study should be interpreted with caution.

There are some important limitations to note of the current study. First, this is a cross-sectional database study design, which allows only for a statistical association between variables, rather than strong evidence of causality, to be determined. Second, the Medical Expenditure Panel Survey data are self-reported by participants over the data collection period, which can lead to recall and other biases. Third, while this study focused on a nationally representative sample of United States adults with pain, given the nature of the United States healthcare system, the findings may not be generalized beyond this population. Fourth, it is important to note that the individuals in this study all had some level of pain, yet it is not clear how much of an influence pain had in the analysis. That is, would these individuals still have used these health services if they did not have pain? Further work is needed to distinguish healthcare service utilization among a similar sample of individuals without pain, or the general population, to determine how the findings compare. Given the number of outcomes and covariates analyzed in this study, there is a possible risk of Type I error inflation. Readers may want to consider a lower statistical significance threshold, e.g., applying a Bonferroni correction, to account for this. Finally, the c–statistics for some of the models were below 0.70 (e.g., emergency room visits and outpatient visits in the multivariable models), indicating limited discriminatory power. Future research, perhaps using a different dataset with additional variables, should consider how to improve the discriminating power of the final models. Future work could also explore the association of additional variables from other databases with health service utilization among United States adults with pain. For example, variables related to medication use or health service access/barriers could be investigated.

## 5. Conclusions

This database analysis found that higher levels of pain intensity were often associated with increased health service utilization for emergency room, inpatient, and office visits. Additional demographic variables were also observed to be associated with some of the health service utilization variables and may be interesting targets for future study. These findings may help researchers, clinicians, and policy makers better understand the associations between pain and other demographic variables with health service use. Further work is warranted to design interventions that may help reduce disparities, optimize health service utilization, and improve health outcomes for United States adults who have pain. The findings of the current study may also help inform future intervention studies or predictive models. 

## Figures and Tables

**Table 1:** Comparison of characteristics among United States adults in the weighted study population organized by pain intensity.

Variable	Extreme Pain Percentage [95% Confidence Interval]	Quite a Bit of Pain Percentage [95% Confidence Interval]	Moderate Pain Percentage [95% Confidence Interval]	Little Pain Percentage [95% Confidence Interval]	*p*-Value
Age					<0.0001
≥70 years	24.1 [18.3–29.8]	31.5 [27.4–35.5]	29.0 [25.8–32.1]	21.1 [19.5–22.8]	
60–69 years	24.4 [19.0–29.7]	24.2 [20.9–27.4]	21.8 [19.3–24.2]	20.4 [18.7–22.2]	
50–59 years	23.6 [18.4–28.9]	21.8 [17.9–25.7]	17.1 [14.2–20.1]	18.8 [17.0–20.5]	
40–49 years	14.9 [10.2–19.6]	8.5 [5.9–11.1]	14.1 [11.1–17.0]	16.2 [14.5–17.8]	
30–39 years	6.5 [3.0–10.0]	8.5 [5.6–11.4]	12.4 [9.5–15.3]	13.1 [11.4–14.8]	
18–29 years	6.5 [3.0–10.1]	5.6 [3.5–7.6]	5.7 [3.6–7.7]	10.5 [9.0–11.9]	
Race					0.0678
White	73.8 [67.5–80.1]	80.4 [77.3–83.6]	77.5 [73.8–81.1]	80.3 [78.0–82.5]	
Others	26.2 [19.9–32.5]	19.6 [16.4–22.7]	22.5 [18.9–26.2]	19.7 [17.5–22.0]	
Hispanic					0.3057
Yes	9.6 [6.4–12.7]	10.5 [6.9–14.1]	11.0 [8.4–13.5]	12.5 [10.8–14.3]	
No	90.4 [87.3–93.6]	89.5 [85.9–93.1]	89.0 [86.5–91.6]	87.5 [85.7–89.2]	
Sex					0.0227
Male	40.7 [34.1–47.4]	40.9 [37.1–44.6]	43.4 [40.1–46.7]	46.5 [44.8–48.3]	
Female	59.3 [52.6–65.9]	59.1 [55.4–62.9]	56.6 [53.3–59.9]	53.5 [51.7–55.2]	
Marriage					<0.0001
Married	36.9 [29.6–44.3]	47.1 [43.2–51.0]	51.3 [47.5–55.1]	56.5 [54.5–58.6]	
Not married	63.1 [55.7–70.4]	52.9 [49.0–56.8]	48.7 [44.9–52.5]	43.5 [41.4–45.5]	
Education					<0.0001
≤High school	56.7 [49.6–63.7]	54.5 [50.1–58.9]	44.9 [41.2–48.6]	38.5 [36.2–40.8]	
>High school	43.3 [36.3–50.4]	45.5 [41.1–49.9]	55.1 [51.4–58.8]	61.5 [59.2–63.8]	
Employment					<0.0001
Employed	20.6 [14.8–26.4]	27.1 [23.1–31.0]	48.0 [44.1–51.8]	63.4 [61.2–65.5]	
Unemployed	79.4 [73.6–85.2]	72.9 [69.0–76.9]	52.0 [48.2–55.9]	36.6 [34.5–38.8]	
Income					<0.0001
Low	57.6 [50.1–65.0]	45.9 [41.5–50.2]	36.1 [32.3–40.0]	23.2 [21.2–25.3]	
Middle	28.1 [21.6–34.6]	28.0 [24.0–31.9]	27.3 [23.8–30.7]	28.9 [26.5–31.4]	
High	14.3 [8.9–19.7]	26.2 [22.1–30.2]	36.6 [32.8–40.4]	47.8 [45.0–50.7]	
Insurance					<0.0001
Private	30.6 [24.5–36.6]	47.2 [42.7–51.6]	55.4 [51.5–59.3]	66.9 [64.6–69.1]	
Public	65.4 [59.0–71.8]	50.0 [45.7–54.3]	39.5 [36.1–43.0]	27.3 [25.5–29.2]	
None	4.0 [1.3–6.7]	2.8 [1.0–4.6]	5.0 [2.8–7.2]	5.8 [4.5–7.0]	
Number of chronic conditions					<0.0001
0–1	12.9 [8.5–17.3]	17.6 [14.0–21.3]	27.1 [23.8–30.4]	42.2 [40.0–44.5]	
2–3	37.6 [30.9–44.2]	32.7 [29.2–36.1]	38.1 [34.3–42.0]	35.9 [34.0–37.8]	
4–5	30.7 [25.3–36.1]	30.3 [26.9–33.6]	24.0 [21.1–27.0]	16.8 [15.2–18.4]	
6+	18.8 [13.3–24.4]	19.5 [16.4–22.5]	10.7 [8.8–12.7]	5.1 [4.2–6.0]	
Any limitation					<0.0001
Yes	84.1 [79.0–89.2]	77.1 [73.2–80.9]	50.8 [47.4–54.1]	26.9 [25.0–28.8]	
No	15.9 [10.8–21.0]	22.9 [19.1–26.8]	49.2 [45.9–52.6]	73.1 [71.2–75.0]	
Exercise					<0.0001
Yes	25.1 [19.4–30.7]	33.0 [29.1–36.9]	45.3 [41.7–48.9]	50.2 [47.8–52.6]	
No	74.9 [69.3–80.6]	67.0 [63.1–70.9]	54.7 [51.1–58.3]	49.8 [47.4–52.2]	
Current smoker					<0.0001
Yes	24.8 [18.8–30.8]	17.9 [14.7–21.0]	18.5 [15.3–21.6]	11.6 [10.3–12.9]	
No	75.2 [69.2–81.2]	82.1 [79.0–85.3]	81.5 [78.4–84.7]	88.4 [87.1–89.7]	
Health					<0.0001
Poor/fair	69.1 [63.0–75.3]	53.0 [49.1–56.9]	27.1 [23.6–30.6]	14.2 [12.6–15.7]	
Good	22.2 [16.6–27.9]	32.0 [28.7–35.4]	42.4 [38.3–46.5]	37.0 [35.0–39.0]	
Very good/excellent	8.7 [5.0–12.3]	15.0 [12.3–17.6]	30.5 [27.0–34.1]	48.9 [46.6–51.1]	
Mental health					<0.0001
Poor/fair	42.3 [35.4–49.2]	28.3 [24.6–32.0]	18.9 [16.0–21.7]	11.8 [10.2–13.3]	
Good	31.2 [25.4–37.0]	39.2 [35.3–43.0]	40.0 [36.1–43.8]	34.4 [32.3–36.4]	
Very good/excellent	26.5 [20.7–32.3]	32.5 [28.3–36.7]	41.2 [37.4–45.0]	53.9 [51.6–56.2]	

Statistical assessments between pain intensity levels for each variable was assessed with chi–squared tests.

**Table 2:** Associations of pain intensity with health service utilization (emergency room visits, inpatient discharges, office visits, outpatient visits) among United States adults with pain in univariate logistic regression analyses.

Pain	Emergency Room Visits Odds Ratio [95% Confidence Interval]	Inpatient Discharges Odds Ratio [95% Confidence Interval]	Office Visits Odds Ratio [95% Confidence Interval]	Outpatient Visits Odds Ratio [95% Confidence Interval]
Extreme	**3.34 [2.57–4.33]**	**4.39 [3.24–5.94]**	**1.97 [1.18–3.30]**	**1.91 [1.45–2.51]**
Quite a bit	**2.91 [2.33–3.64]**	**3.28 [2.52–4.28]**	**1.99 [1.39–2.85]**	**1.44 [1.17–1.78]**
Moderate	**1.61 [1.31–1.97]**	1.27 [0.97–1.66]	1.34 [1.00–1.80]	**1.26 [1.07–1.49]**
Little	Reference	Reference	Reference	Reference

Statistically significant findings are reported in bold. C–statistics: emergency room visits = 0.610; inpatient discharges = 0.617; office visits = 0.554; outpatient visits = 0.544. All Wald statistics < 0.0001.

**Table 3:** Associations of pain intensity and other demographic variables with health service utilization (emergency room visits, inpatient discharges, office visits, outpatient visits) among United States adults with pain in multivariable logistic regression analyses.

Variable	Emergency Room Visits Odds Ratio [95% Confidence Interval]	Inpatient Discharges Odds Ratio [95% Confidence Interval]	Office Visits Odds Ratio [95% Confidence Interval]	Outpatient Visits Odds Ratio [95% Confidence Interval]
Pain				
Extreme	**1.72 [1.27–2.33]**	**2.10 [1.44–3.08]**	1.65 [0.95–2.89]	1.24 [0.91–1.69]
Quite a bit	**1.75 [1.37–2.24]**	**1.66 [1.21–2.28]**	**1.47 [1.03–2.11]**	0.89 [0.71–1.11]
Moderate	**1.28 [1.02–1.60]**	0.91 [0.67–1.25]	1.17 [0.84–1.63]	1.04 [0.87–1.24]
Little	Reference	Reference	Reference	Reference
Age				
≥70 years	**0.59 [0.40–0.89]**	**1.86 [1.04–3.32]**	**3.16 [1.77–5.63]**	**1.79 [1.18–2.72]**
60–69 years	**0.53 [0.36–0.79]**	1.40 [0.78–2.53]	1.04 [0.60–1.80]	1.45 [0.97–2.18]
50–59 years	**0.56 [0.37–0.85]**	1.01 [0.56–1.83]	0.88 [0.54–1.42]	1.32 [0.90–1.95]
40–49 years	0.69 [0.45–1.06]	1.27 [0.71–2.30]	1.37 [0.73–2.56]	1.29 [0.86–1.95]
30–39 years	0.66 [0.42–1.06]	1.24 [0.67–2.33]	0.79 [0.48–1.32]	1.15 [0.76–1.75]
18–29 years	Reference	Reference	Reference	Reference
Race				
White	0.96 [0.77–1.20]	0.98 [0.74–1.29]	**1.38 [1.03–1.86]**	1.13 [0.92–1.37]
Others	Reference	Reference	Reference	Reference
Hispanic				
Yes	1.06 [0.79–1.43]	0.92 [0.64–1.31]	**0.61 [0.45–0.83]**	**0.75 [0.58–0.98]**
No	Reference	Reference	Reference	Reference
Sex				
Male	0.90 [0.73–1.09]	0.81 [0.64–1.01]	**0.56 [0.43–0.73]**	**0.69 [0.59–0.80]**
Female	Reference	Reference	Reference	Reference
Marriage				
Married	0.89 [0.74–1.08]	1.14 [0.93–1.40]	1.29 [0.94–1.76]	0.99 [0.85–1.16]
Not married	Reference	Reference	Reference	Reference
Education				
≤High school	1.09 [0.91–1.30]	1.24 [1.00–1.54]	**0.48 [0.37–0.64]**	**0.78 [0.67–0.91]**
>High school	Reference	Reference	Reference	Reference
Employment				
Employed	1.26 [1.00–1.58]	1.13 [0.87–1.48]	**1.52 [1.13–2.05]**	1.02 [0.83–1.24]
Unemployed	Reference	Reference	Reference	Reference
Income				
Low	1.21 [0.91–1.60]	1.27 [0.90–1.78]	**0.55 [0.38–0.81]**	**0.69 [0.55–0.86]**
Middle	1.01 [0.81–1.26]	1.14 [0.87–1.50]	0.79 [0.54–1.15]	**0.77 [0.64–0.93]**
High	Reference	Reference	Reference	Reference
Insurance				
Private	**1.80 [1.08–3.00]**	**3.80 [1.71–8.47]**	**3.47 [2.30–5.22]**	**3.64 [2.15–6.17]**
Public	**1.96 [1.16–3.31]**	**3.83 [1.67–8.78]**	**3.36 [2.08–5.44]**	**3.46 [2.04–5.88]**
None	Reference	Reference	Reference	Reference
Number of chronic conditions				
0–1	**0.48 [0.36–0.65]**	**0.48 [0.33–0.70]**	**0.42 [0.19–0.93]**	**0.47 [0.35–0.63]**
2–3	**0.57 [0.45–0.73]**	**0.51 [0.39–0.68]**	0.90 [0.43–1.91]	**0.67 [0.53–0.84]**
4–5	**0.73 [0.57–0.93]**	**0.64 [0.49–0.84]**	2.03 [0.96–4.29]	**0.76 [0.59–0.96]**
6+	Reference	Reference	Reference	Reference
Any limitation				
Yes	**1.40 [1.15–1.71]**	**1.45 [1.11–1.90]**	**1.83 [1.32–2.54]**	**1.57 [1.32–1.88]**
No	Reference	Reference	Reference	Reference
Exercise				
Yes	0.86 [0.71–1.04]	0.89 [0.72–1.10]	1.00 [0.76–1.32]	0.93 [0.81–1.07]
No	Reference	Reference	Reference	Reference
Current smoker				
Yes	**1.31 [1.04–1.65]**	0.86 [0.64–1.14]	**0.64 [0.47–0.87]**	**0.71 [0.57–0.89]**
No	Reference	Reference	Reference	Reference
Health				
Poor/fair	**1.92 [1.45–2.53]**	**2.41 [1.72–3.38]**	1.32 [0.90–1.95]	**1.53 [1.22–1.92]**
Good	**1.32 [1.03–1.70]**	1.02 [0.77–1.35]	**1.73 [1.23–2.45]**	**1.23 [1.01–1.49]**
Very good/excellent	Reference	Reference	Reference	Reference
Mental health				
Poor/fair	0.84 [0.66–1.08]	**0.67 [0.48–0.94]**	1.08 [0.74–1.59]	1.01 [0.81–1.27]
Good	0.98 [0.79–1.20]	0.87 [0.68–1.12]	0.91 [0.66–1.24]	0.94 [0.79–1.12]
Very good/excellent	Reference	Reference	Reference	Reference

Statistically significant findings are reported in bold. C–statistics: emergency room visits = 0.685; inpatient discharges = 0.711; office visits = 0.784; outpatient visits = 0.671. All Wald statistics < 0.0001.

## Data Availability

Dataset is available on request from the authors. The raw data supporting the conclusions of this article will be made available by the authors on request.

## Appendix A

**Table A1:** Description of the variables included in the analysis.

Variable Text	Variable Response Options	Medical Expenditure Panel Survey Variable Name
During the past 4 weeks, how much did pain interfere with your normal work (including both work outside the home and housework)?	Cannot be computed Inapplicable Not at all A little bit Moderately Quite a bit Extremely	ADPAIN42
Number of office–based provider visits 2021	Count data	OBTOTV21
Number of outpatient department provider visits 2021		OPTOTV21
Number of emergency rooms visits 2021		ERTOT21
Number of hospital discharges 2021		IPDIS21
Age as of December 31 2021	Inapplicable 0–85 (top coded as 85)	AGE21X
Race	White—no other race reported Black—no other race reported American Indian/Alaska Native—no other race reported Asian/Native Hawaiian/Pacific Islander—no other race reported Multiple races reported	RACEV1X
Hispanic	Hispanic Not Hispanic	HISPANX
Sex	Male Female	SEX
Marital status as of December 31 2021	Don’t know Refused Married Widowed Divorced Separated Never married Under age 16—inapplicable	MARRY21X
Years of education when first entered MEPS	Cannot be computed Don’t know Refused Inapplicable No school/kindergarten only Elementary grades 1–8 High school grades 9–11 Grade 12 1 year college 2 years college 3 years college 4 years college 5+ years college	EDUCYR
Employment status	Cannot be computed Don’t know Refused Inapplicable Employed Job to return to Got a job Not employed	EMPST31/42/53
Family income as percentage of poverty line	Poor/negative Near poor Low income Middle income High income	POVCAT21
Health insurance coverage indicator	Any private Public only Uninsured	INSCOV21
Multiple diagnoses of high blood pressure	Cannot be computed Don’t know Refused Inapplicable Yes No	BPMLDX
Coronary heart disease diagnosis		CHDDX
Angina diagnosis		ANGIDX
Heart attack (MI) diagnosis		MIDX
Other heart disease diagnosis		OHRTDX
Stroke diagnosis		STRKDX
Emphysema diagnosis		EMPHDX
Chronic bronchitis in the last 12 months		CHBRON31
High cholesterol diagnosis		CHOLDX
Cancer diagnosis		CANCERDX
Diabetes diagnosis		DIABDX_M18
Joint pain in the last 12 months		JTPAIN31_M18
Arthritis diagnosis		ARTHDX
Asthma diagnosis		ASTHDX
Any limitation in 2021	Cannot be computed Inapplicable Yes No	ANYLMI21
Moderate/vigorous exercise for at least 30 min at least five times per week	Don’t know Refused Inapplicable Yes No	PHYEXE53
How often smoke cigarettes	Don’t know Refused Inapplicable Every day Some days Not at all	OFTSMK53
Perceived health status	Don’t know Refused Inapplicable Excellent Very good Good Fair Poor	RTHLTH31/42/53
Perceived mental health status		MNHLTH31/42/53
